# Immunotoxicity of nanomaterials in health and disease: Current challenges and emerging approaches for identifying immune modifiers in susceptible populations

**DOI:** 10.1002/wnan.1804

**Published:** 2022-11-23

**Authors:** Sabine Hofer, Norbert Hofstätter, Benjamin Punz, Ingrid Hasenkopf, Litty Johnson, Martin Himly

**Affiliations:** ^1^ Division of Allergy & Immunology, Department of Biosciences & Medical Biology Paris Lodron University of Salzburg Salzburg Austria

**Keywords:** adverse outcome pathways, complex models, data FAIRness, diseased state, pre‐existing conditions

## Abstract

Nanosafety assessment has experienced an intense era of research during the past decades driven by a vivid interest of regulators, industry, and society. Toxicological assays based on in vitro cellular models have undergone an evolution from experimentation using nanoparticulate systems on singular epithelial cell models to employing advanced complex models more realistically mimicking the respective body barriers for analyzing their capacity to alter the immune state of exposed individuals. During this phase, a number of lessons were learned. We have thus arrived at a state where the next chapters have to be opened, pursuing the following objectives: (1) to elucidate underlying mechanisms, (2) to address effects on vulnerable groups, (3) to test material mixtures, and (4) to use realistic doses on (5) sophisticated models. Moreover, data reproducibility has become a significant demand. In this context, we studied the emerging concept of adverse outcome pathways (AOPs) from the perspective of immune activation and modulation resulting in pro‐inflammatory versus tolerogenic responses. When considering the interaction of nanomaterials with biological systems, protein corona formation represents the relevant molecular initiating event (e.g., by potential alterations of nanomaterial‐adsorbed proteins). Using this as an example, we illustrate how integrated experimental–computational workflows combining in vitro assays with in silico models aid in data enrichment and upon comprehensive ontology‐annotated (meta)data upload to online repositories assure FAIRness (Findability, Accessibility, Interoperability, Reusability). Such digital twinning may, in future, assist in early‐stage decision‐making during therapeutic development, and hence, promote safe‐by‐design innovation in nanomedicine. Moreover, it may, in combination with in silico‐based exposure‐relevant dose‐finding, serve for risk monitoring in particularly loaded areas, for example, workplaces, taking into account pre‐existing health conditions.

This article is categorized under:

Toxicology and Regulatory Issues in Nanomedicine > Toxicology of Nanomaterials

## Graphical abstract

Investigating the immunotoxicity of nanomaterials in humans demands an overlay of relevant entities of the immune system (level 1) with a perspective on pathways from molecular initiation via key events to clinical outcome (level 2) and further modification by pre‐existing conditions to be able to mimic differently susceptible individuals (level 3).

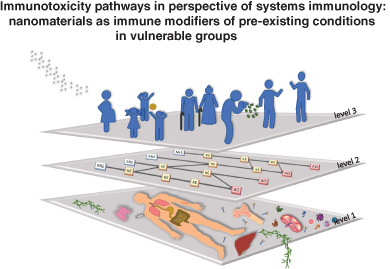

## 1 INTRODUCTION

Investigating the safety of nanomaterials (NMs) has been a major theme of the past European framework programs. For instance, during FP7, the EU's 2007–2013 research funding program, 48 collaborative projects on NM toxicity, exposure monitoring, risk management, and regulation, were supported by a total of EUR 177 million in EU funding (Sealy, 2015). This effort has continued under Horizon 2020, and within Horizon Europe currently takes its transition to Safety‐and‐Sustainability‐by‐Design (SSbD) strategies for innovation in advanced materials, micro‐/nanoplastics, and nanomedicine (NanoSafety Cluster Working Group (NSC), 2022) in line with the European Union's Chemical Strategy for Sustainability and the Green Deal initiative (EC, 2019, 2020). Basic research endeavored to elucidate the underlying mechanisms of how NMs may impact human health and the environment, while regulators, in combination with exposure assessment, attempted to deduce safety margins for risk mitigation. Historically, the toxicological evaluation of NMs has been driven by phenomenological strategies, but it became apparent that there still remain major shortcomings that could not be fully addressed in this way. Hence, we have emerged at an era adopting advanced in vitro and in silico methods for supplementing well‐established experimental systems. Besides, the re‐use of data deploying in silico models may provide new insights based on existing data to strengthen the understanding of interactions, as particularly of importance in immunotoxicity assessment. Yet, new emerging and more tightly integrated pathways are far from being “ready to go” and able to address all obstacles to immunotoxicity evaluation. Most critically, these advancements do not help to clarify one of the most pressing shortcomings in NM toxicology testing, which concerns the question, whether a specific dose at any point of an experimental system or mechanistic adverse outcome pathway (AOP) is reasonably justified, for example, as being a physiological response in vivo and linked to a real‐world exposure scenario.

The immune system has long ago been recognized as a complex system, and not surprising, for its investigation, the term systems immunology has been coined meanwhile (Davis et al., 2017). So far, the immune systems' many entities have been studied in a somewhat isolated mode (i.e., examining parts, but not taking into account activities across the entire system), however, it is obvious that 350 clusters of differentiation (CD) antigens on the cells' surfaces, over 100 cytokines and chemokines, at least as many cell subsets, and thousands of genes make a more systemic view essential for understanding the interplay between them. Moreover, an eminent characteristic of the immune system's complexity lies in its spatially distributed functionality. This, in fact, results in a vast divergence between local and remote effects, essentially demanding a more distant, well‐overviewing perspective for understanding multiple stimulus‐effect relationships combined with knowledge derived from advanced models of ever‐growing complexity (e.g., to mimic cell–cell interactions within microenvironments, etc.).

**FIGURE 1:**
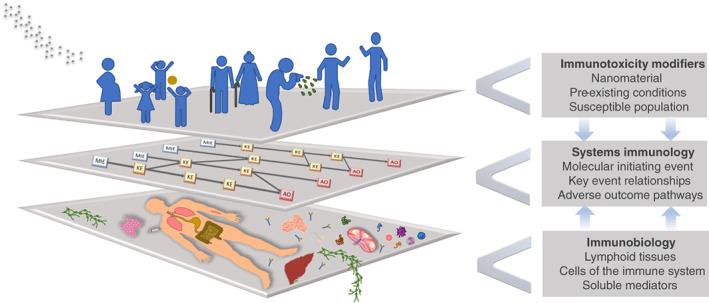
Immunotoxicity networks link bio–nano interactions at the molecular level through adverse outcome pathways to their clinical presentation (in the middle). Systems immunology is a discipline that endeavors to crosslink the relevant entities of the immune system (at the bottom) with susceptible populations (children, elderly, pregnant, immunocompromised, sensitized) and NMs as immune modifiers, taking pre‐existing conditions into account (at the top).

In this review, we will overview the current conceptual landscape in immunotoxicity assessment (such as the emerging concept of AOPs) toward NMs and relate it to the specific challenges that studying the immune system, a highly complex system owing to its well‐organized, spatially distributed and time‐resolved processes, brings along. We will, therefore, illustrate a number of pre‐existing states of susceptible populations within the human “study cohort” to lay out the specific conditions (immunotoxicity modifiers) presented by vulnerable groups, while acknowledging the impact of NMs itself representing such a modifier. The discussion of the AOP concept for NMs will include a presentation of protein corona formation as domain‐characteristic molecular initiating event, and, as indicated and exemplified for this case, present a way to combine experimental research with computational modeling. We will, furthermore, measure the emerging AOP concept as a systems immunology approach for its suitability to the presented immunotoxicity modifiers and close with recommendations for advanced experimental models in need (Figure 1).

## 2 ADVERSE OUTCOME PATHWAYS AS AN EMERGING CONCEPT FOR IMMUNOTOXICOLOGY OF NANOMATERIALS

The evolution of nanotechnology has led to the generation of novel concepts aiding the development of innovative nanomedical platforms. However, most of these developments are hindered due to the unidentified detrimental effects of NMs, which also include the safety concerns of nanoparticles (NPs) in consumer products and upon occupational or environmental exposure (Zielińska et al., 2020). Some of the important and promising ways to assess nanosafety involve the evaluation of cytotoxicity, genotoxicity, immunotoxicity, oxidative stress, and inflammation (Dusinska et al., 2017). Immunotoxicity evaluation of NMs is not only crucial for the safety assessment but also for the market authorization of nanomedical products. As of now, the nonclinical regulatory immunotoxicity testing of nonbiological nanotherapeutics is carried out by following the International Council for Harmonization (ICH) of Technical Requirements for Pharmaceuticals for Human Use guideline S8 (EMA, 2006). According to this guideline, immunotoxicity evaluation involves standard in vivo toxicity studies. Even though in vivo models give a complete pharmacokinetics and pharmacodynamics profile they often fail in predicting toxicity in humans (Van Norman, 2019). Furthermore, they are costly, laborious and do not go in line with the efforts by the government regulatory agencies/scientific organizations in reducing animal experiments. Thus, a cost‐effective alternative to animal experiments would be in vitro human cell line‐based toxicity testing. The most common techniques for in vitro immunotoxicity evaluation involve enzyme‐linked immunosorbent assay (ELISA), quantitative reverse transcription‐polymerase chain reaction (qRT‐PCR), flow cytometry, transcriptomics, multiplexing, and repertoire sequencing to determine, for example, antibody titers, cytokine/chemokine expression, and secretion levels, upregulation of surface markers, T cell profiling (Corsini & House, 2018). The United States' Academy of Sciences visualizes the transition to in vitro toxicity pathway assays as a possibility to improve the efficiency of toxicity testing (Krewski et al., 2010). The AOP concept utilizes this transition where cellular response pathways are influenced by an initiating event leading to adverse health outcomes. It was first described for the ecological risk assessment of chemicals. This work portrayed the application of AOPs in the toxicity evaluation (Ankley et al., 2010). The concept of AOPs combines the pre‐existing data with in vitro, in chemico, and in silico data to get a meaningful conclusion, thereby saving time and sparing animal experimentation. An AOP is established by describing how a biological event at the molecular level, termed a molecular initiating event (MIE) resulting from encounter with a stressor, for example, a material/compound, can generate a series of key events (KEs) finally leading to an adverse outcome (AO). A MIE represents the initial major event in an AOP that initiates the pathway. The MIEs can be specific or nonspecific, that is, being based on a specific interaction of a chemical moiety with a particular receptor or on nonspecific interactions with cellular proteins (Tirumala et al., 2021). In the case of NPs, the induction of toxicity is mostly through nonspecific mechanisms. One such nonspecific interaction represents the formation of a bio‐corona at the NP surface, which will be discussed in detail in the next chapter. The physicochemical properties of NMs are altered based on their exposure to biomolecules like proteins, lipids, carbohydrates, and more in the human body or environment. This could eventually affect the fate and biological impact of NPs by triggering an immune response eventually altering their immunotoxicity profiles (Westmeier et al., 2016). The KEs in an AOP define changes in the physiological state that occur at a cellular, tissue, organ, and organism level. Key event relationships (KERs) represent the connections among the different KEs (Villeneuve et al., 2014).

The concept of AOP is relevant in the nano community both for the development of strategies for the evaluation of immunotoxicity and identification of hazards for human health. However, there are certain challenges to its initiation. The adverse immune outcomes from NMs are not fully identified and it is very difficult to identify potential KEs from the reported biological endpoints (Halappanavar et al., 2020). The major KEs that could be identified from immunotoxicity studies of NPs include the induction of oxidative stress, inflammation, and cytotoxicity in immune cells (B. M. Johnson et al., 2015; Mendoza et al., 2014). At present, out of close to 400 AOPs in the AOP‐Wiki, the community‐curated collection (https://aopwiki.org), only 11 elucidate the role of a NM as a stressor, and the majority of these AOPs are in their developmental stage (Table 1). Thus, there is a necessity to identify novel KEs that could potentially lead to adverse immune effects. The KEs described below in more detail are based on established KEs in the AOP‐Wiki and can be linked to NM‐induced immunotoxic effects by scientific evidence.

**TABLE 1:** AOPs with NM as stressor retrieved from AOP‐Wiki repository

AOP ID	Stressor	MIE	KEs	Outcome	Reference
144	Nanoparticles	Endocytic lysosomal uptake	Lysosomal disruption Mitochondrial dysfunction Cell death/injury Increased inflammatory mediators Leukocyte recruitment HSC activation ECM alteration	Liver fibrosis	(F. Wang et al., 2013) (Cui et al., 2011) (Alarifi et al., 2013)
392	Nanoparticles	Decreased fibrinolysis, Activated bradykinin	Increased pro‐inflammatory mediators Increased recruitment of inflammatory cells	Hyperinflammation	(Ekdahl et al., 2018) (Ekstrand‐Hammarström et al., 2015)
207	Silver nanoparticles	NADPH oxidase activation	ROS formation Mitochondrial damage Oxidative stress hypoxia‐inducible factor 1 activation DNA damage repair Apoptosis	Reproductive failure	(Jeong et al., 2018)
208	UV activated titanium dioxide nanoparticles		JAK/STAT pathway activation TGF beta pathway activation	Reproductive failure	(H. Kim et al., 2017)
209	Silica nanoparticles		Unsaturated fatty acid upregulation Glutathione synthesis Perturbation of cholesterol Glutathione homeostasis	Hepatotoxicity	(Chatterjee et al., 2018)
210	Graphene oxide nanoparticles		Oxidative stress c‐Jun N‐terminal kinase activation Transcription factor FOXO activation Wnt signaling pathway inhibition Embryogenesis defect	Reproductive failure	(Y. Kim et al., 2018)
237	Graphene oxide nanoparticles/insoluble nano‐sized particles	Sensing of the stressor by pulmonary cells	Increased production of pulmonary, pro‐inflammatory cytokines Increased production of pulmonary serum amyloid A (SAA) Formation of HDL‐SAA Increased systemic total cholesterol pool Foam cell formation	Plaque progression in arteries	(Poulsen et al., 2015)
409	Multi‐walled carbon nanotubes and carbon nanotubes	Frustrated phagocytosis	Increased inflammatory cell recruitment Increased pro‐inflammatory mediator's secretion Increased reactive oxygen species Increased DNA damage and mutation Genomic instability Increased cell proliferation	Increased mesotheliomas	(M. S. Boyles et al., 2015) (Chernova et al., 2017)
241	Carbon nanotubes	TGF beta1 activation	Increased differentiation of fibroblasts Epithelial–mesenchymal transition induction Collagen accumulation TGF beta pathway activation	Pulmonary fibrosis	(Vietti et al., 2016)
173	Carbon nanotubes/ carbon nanofibers	Interaction with the lung resident cell membrane components	Increased pro‐inflammatory mediators Increased recruitment of inflammatory cells Loss of alveolar‐capillary membrane integrity Increased activation of Th2 cells Increased fibroblast proliferation and myofibroblast differentiation Increased extracellular matrix deposition	Lung fibrosis	(Dong & Ma, 2016)
319	Cationic polyamidoamine dendrimer	ACE2 inhibition	Increased angiotensin II Increased plasma Angiotensin II Collagen accumulation	Lung fibrosis	(Sun et al., 2015)

### 2.1 KE1: Activation of the complement system

Complement activation could be considered as a potential KE. The excessive activation of the complement system can contribute to the development of inflammation, allergy, and complement activation‐related pseudoallergy (CARPA; Stater et al., 2021). An excessive unconstrained activation of the complement could even have fatal consequences in humans, for example, through anaphylactic events. CARPA is a non‐IgE‐mediated hypersensitivity reaction where the anaphylatoxins C3a and C5a bind to the mast cells resulting in the release of vasoactive mediators (Macdougall & Vernon, 2017). Even the highly biocompatible and biodegradable NMs like poly‐lactic‐co‐glycolic acid (PLGA) have been reported to activate the complement system (Fornaguera et al., 2015). Furthermore, Szebeni et al. highlighted the importance of testing the activation of complement system in particulate systems as they found the induction of CARPA in clinically approved liposomal formulations (Szebeni & Moghimi, 2009). Actual relevance of such mechanisms is provided by adverse reactions against PEGylated liposome carriers of anti‐SARS‐CoV‐2 mRNA vaccines (Brockow et al., 2021).

### 2.2 KE2: NLRP3 Inflammasome activation

Activation of the NOD‐like receptor family pyrin domain containing 3 (NLRP3) inflammasome is a critical step in the inflammatory cascade resulting in a pro‐inflammatory response. This can prompt the release of cytokines such as IL‐1β through caspase 1 giving rise to cell death (pyroptosis). NPs have been widely reported to induce NLRP3 inflammasome activation (Baron et al., 2015). This response is actually beneficial for vaccine delivery especially as an adjuvant. The most commonly applied adjuvant aluminum hydroxide (alum) acts by the secretion of the pro‐inflammatory cytokines IL‐1β and IL‐18, thereby activating the NLRP3 inflammasome (Ivanov et al., 2020). However, the intravenous administration of a nanoparticulate drug delivery system with a pro‐inflammatory immune response can result in systemic side effects, thus, exhibit immunotoxicity. Therefore, it is crucial to study the inflammasome activation of the particulate delivery systems. Boyles et al. demonstrated that chitosan‐functionalized gold NPs cause cytotoxicity in the human monocytic cell line THP‐1 by inducing strong pro‐inflammatory conditions (Boyles, Kristl, et al., 2015; Boyles, Young, et al., 2015). In line with this, Zhang et al. reported the activation of NLRP3 inflammasome in hepatocytes leading to pyroptosis indicating lung inflammation and hepatotoxicity (Zhang et al., 2018). The aberrant activation of inflammasome has been reported to contribute to the production of pro‐inflammatory cytokines (IL‐1β, IL‐18) leading to signal transduction, resulting in the progression of cardiovascular diseases (D. Liu et al., 2017). Thus, aberrant activation of inflammasome by NPs can clinically contribute to adverse outcomes including lung fibrosis (Zheng et al., 2018), emphysema, and cardiovascular diseases (Chen et al., 2017; Yuan et al., 2019).

### 2.3 KE3: Dendritic cell maturation

Dendritic cells (DCs) are key players in the immune system. DCs can encounter NPs in the tissues like skin, lining of nose, lungs, stomach and blood, endocytose them, and become activated in a process termed DC maturation. The endocytosis of NPs can lead to the maturation of DCs, which is characterized by the expression of costimulatory molecules and cytokine release. After maturation, DCs migrate to the lymph node where they can further activate naïve T cells and lead to their polarization. Thus, maturation of DCs is also an indication of immunomodulatory effects (Jia et al., 2018) transmitted via surface molecules such as CD40, CD80, CD86, and MHC II. This maturation of DCs by NPs can have both beneficial and detrimental effects including the alteration of immune response by eliciting an autoimmune disorder or hypersensitivity reaction or allowing potential application as an adjuvant in vaccine (Ganguly et al., 2013). In case of allergy, the mature DCs present the allergen to naïve T cells leading to their proliferation and differentiation to a T helper 2 type (L. Johnson et al., 2020). Carbon nanotubes, silica, and silver NPs have been shown to induce hypersensitivity reactions in mice when co‐exposed to allergens (Chuang et al., 2013; Hirai et al., 2012; Nygaard et al., 2009). Furthermore, the United States' Food and Drug Administration (US FDA) have published a safety warning for Ferumoxytol (Feraheme®), a supermagnetic iron oxide NP coated with low molecular weight semi‐synthetic carbohydrate utilized for iron replacement therapy, to cause severe life‐threatening hypersensitivity reactions. However, the exact mechanism of induction of the hypersensitivity reaction is unclear (USFDA, 2015) and could be a class problem of approved colloidal iron intravenous supplementation (Wetmore et al., 2017). In contrast, NMs have been described to result in immunological intolerance to self‐antigen, thereby leading to autoimmune disorders. Mohamed et al. have shown that single‐walled carbon nanotubes cause protein citrullination in cultured human cells and mouse lung tissue. Posttranslational citrullination of proteins induces significant biochemical or conformational changes leading to recognition as non‐self by the immune system causing an autoimmune response like rheumatoid arthritis (Mohamed et al., 2012). In summary, the maturation of DCs by NPs contributes to hypersensitivity reactions and autoimmune disorders of diverse clinical presentation.

Altogether, the emerging concept of AOPs can be applied well for the evaluation of immunotoxicity of NMs. After having discussed KEs that may be relevant for NM, it is important to elucidate the mechanisms of how such KEs are initiated at a molecular level.

## 3 PROTEIN CORONA FORMATION AS A GENUINE NANOMATERIAL‐SPECIFIC MIE

**FIGURE 2:**
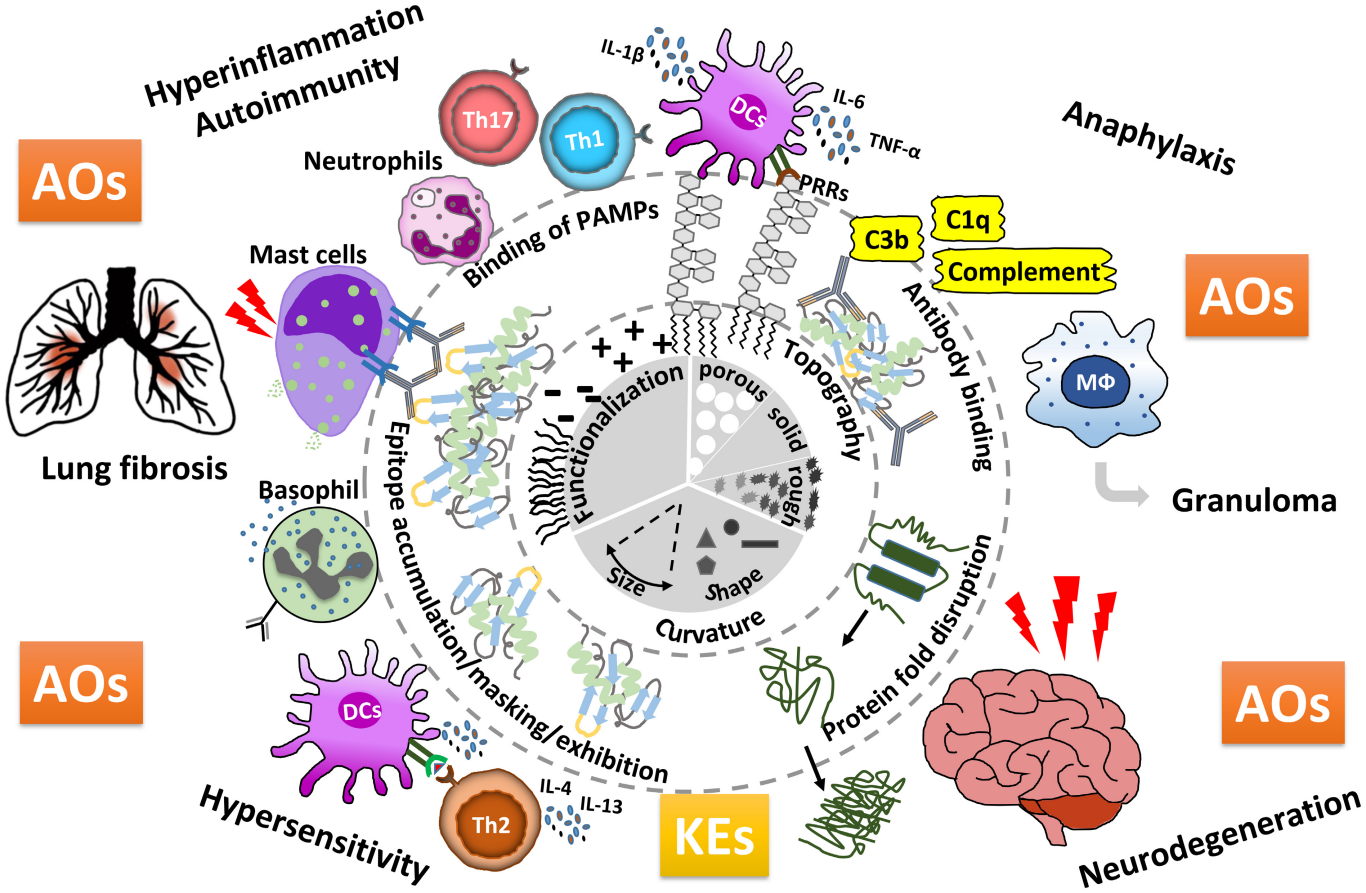
Protein corona formation as a genuine NM‐specific MIE depends on NM‐intrinsic properties such as curvature, topography, and functionalization (displayed within the inner broken line) causing binding of PAMPs, oriented antigens with epitopes accumulated, masked, or, exhibited, or antibodies, as well as fold disruption of bound protein (displayed between the broken lines). These MIEs lead to KEs such as protein aggregation/fibrillation, complement activation, DC maturation, T cell activation/polarization, pro‐inflammatory mediator release, granulocyte/macrophage infiltration, mast cell/basophil degranulation, and so forth resulting in different adverse outcomes (AOs).

A MIE is an initial interaction between a molecule/foreign compound and a biomolecule or biosystem that can be causally linked to an outcome via a pathway (Allen et al., 2014). This also holds true for NMs appearing as stressors that initiate molecular events within organisms at the cells' surfaces, and a number of MIEs have been related to NPs, such as disruption of lung surfactant functionality, lysosomal destabilization (upon being taken up), oxidation of cell membrane, interaction with cell membrane components (e.g., with toll‐like receptors) or frustrated phagocytosis (Halappanavar et al., 2020). Upon entry of NPs into a biological fluid, the present proteins will immediately associate and coat the NPs, depending on affinity, abundance, and time of interaction, to form the so‐called protein corona (Casals et al., 2010). As a result, further recognition and interaction with biological systems are determined by the protein corona, which provides the biological identity of the NP, a fact which has been previously recognized to impact the fate of NPs within organisms and the environment (Klein, 2007; Lynch et al., 2007; Westmeier et al., 2016). The adsorbed proteins may, however, undergo conformational alterations, expose novel structures (neoepitopes) or arrange in specific orientations resulting in altered accessibility of epitopes for antibody recognition (accumulation vs. hiding/masking), subsequently triggering adverse biological effects (Mills‐Goodlet et al., 2021). Figure 2 highlights protein corona‐induced effects that have a perspective for altering immune function.

The protein corona concept has been described first in the 1960s (Vroman, 1962), and its specific features have been characterized later in more detail (Cedervall et al., 2007), resulting in affinity rates and stoichiometries of association and dissociation of NPs and adsorbed proteins. Later, the process of protein corona formation was differentiated into a “hard protein corona”, understood as the tightly bound layer at the NP surface, while the “soft protein corona” was considered to include the interchangeable, more loosely and reversibly bound layers within the NP‐protein complex (Docter et al., 2015; Monopoli et al., 2012; Tenzer et al., 2013; Walczyk et al., 2010; Walkey et al., 2014). As part of the immune system pathogen‐associated molecular patterns (PAMPs) and damage‐associated molecular patterns (DAMPs) are present as an alarm system for invading pathogens and to resolve damaged tissue. Engineered NPs coated with complex protein structures can act as NM‐associated molecular patterns (NAMPs) and can be detected by pattern recognition receptors (PRRs). Hydrophobicity of the NP surface represents a danger signal recognized by the immune system triggering inflammation and altering adaptive immunity as ex vivo experiments showed (Moyano et al., 2012). Hereby mouse splenocytes showed altered gene expression profiles when exposed to gold NPs. The immune cells were most likely unable to detect the bare surface, but different biocorona compositions can initiate alternative immune patterns. In the context of immunomodulation, it has been shown that when NPs trigger T helper type 1 cells, type 1‐polarized macrophages, and B lymphocytes, the secreted molecules will generate an acute inflammation reaction but no neoplastic events. In contrast, when T helper type 2 cells, type 2‐polarized macrophages, and regulatory T lymphocytes are activated, chronic inflammation is sustained and tumorigenesis becomes more likely (Fadeel, 2012; Farrera & Fadeel, 2015). The formation of protein corona can eventually result in complement activation leading to severe inflammation. The surface nanotopography of NMs has been proved to alter the protein corona formation and thereby change complement activation (Hulander et al., 2011). Gold NPs (58 nm) with a smooth surface, when pre‐adsorbed with human immunoglobulin G (IgG), can bind to the head groups of complement component 1q (C1q) resulting in the activation of the classical pathway of the complement system. However, altering the hydrophobicity of NMs' surface significantly reduced the complement activation. Moreover, opsonins (iC3b, C3b) were found depositing onto the carbon nanotubes' surface resulting in complement activation with the development of granuloma formation due to macrophage infiltration (Salvador‐Morales et al., 2006). The adsorption of nonspecific blood proteins on the NMs' surface can induce conformational changes in the proteins eventually guiding for complement attack (Gbadamosi et al., 2002). NP–protein interaction can trigger a pro‐inflammatory immune response which can contribute to the development of hyper‐inflammation. Adsorption of human serum protein (high‐density lipoprotein and bovine serum albumin, BSA) to silver NPs were shown to induce conformational changes in the structures of bound proteins. This, in fact, altered the cellular uptake of NPs (specifically through scavenger receptors) and initiated cytotoxicity and an inflammatory response (increased mRNA expression of the pro‐inflammatory cytokine IL‐6) in in vitro cellular models (epithelial and endothelial cells; Shannahan et al., 2015). Furthermore, poly‐acrylic acid‐conjugated gold NPs promoted unfolding of adsorbed fibrinogen by exposing the amino acid sequence 377–395 in the C‐terminus of the γ chain. The exposed amino acid was shown to facilitate the interaction with the leukocyte receptor MAC‐1 and triggered the NF‐kB signaling pathway. The activation of NF‐kB signaling pathway further led to the release of pro‐inflammatory cytokines from human monocytic THP‐1 cells (Deng et al., 2011). Similarly, adsorption of BSA to poly(methacrylic acid) nanoporous polymer particles induced conformational alterations resulting in internalization by scavenger receptor‐mediated endocytosis and pro‐inflammatory cytokine secretion in macrophage‐like differentiated THP‐1 cells (Yan et al., 2013). Protein adsorption onto the NM surface can favor conformational changes in the protein, thereby imposing destabilization of the native protein fold, reducing their thermal and pH stability and eventually leading to protein denaturation. Allergens adsorbed onto the surface of amorphous silica NPs have been reported to induce changes in the fold stability, especially in the alpha‐helical structures of Bet v 1 (the major birch pollen allergen), thereby altering the immunological response (L. Johnson et al., 2021), while the formation of the tubulin protein corona on the surface of titanium dioxide (TiO_2_) NPs imposed an inhibitory effect on tubulin polymerization, an important step in cell division. The interaction with NPs altered the folding of both tubulin and microtubule proteins eliciting functional alterations (Gheshlaghi et al., 2008). The misfolding of proteins at the NM surface resulting in the formation of large aggregates or fibrils (protein fibrillation) mechanistically mimics a root cause of neurodegenerative disorders including Parkinson's and Alzheimer's disease. Quantum dots (QDs) of about 12 nm in size, and with a slightly negative charge were experimentally proved to promote fibrillation of human insulin under physiological conditions. However, strongly negatively or less positively charged QDs of the same size, as well as similarly charged larger/smaller‐sized ones, did not exhibit any fibrillation. Hence, the surface charge and size of QDs have a major role in the destabilization of the structure of insulin (Sukhanova et al., 2019). In addition to these observations, covalent conjugation of peptide amyloid beta 40 to fluorescent maghemite magnetic NPs increased the fibrillation process but similar conjugation of Leu‐Pro‐Phe‐Phe‐Asp peptides inhibited the fibrillation (Skaat et al., 2011). Hence, the intrinsic property of proteins and the physicochemical properties of NMs can play a significant role in protein–NP interactions and thus can alter the effects induced.

Summarizing the above‐mentioned studies, it becomes evident that a profound investigation and understanding of protein corona formation (its composition, induced structural changes, epitope [re]arrangements, etc.) is essential to assess the consequences of protein adsorption on immune safety and biocompatibility. However, experimental assays at the nano‐bio interface have their limits in what we can learn from them at the molecular level giving room for developments in computational modeling. We will, therefore, overview the latest advancements from in silico prediction methods in the next chapter proposing combining results from both areas in an interactive manner.

## 4 INVESTIGATING THE IMMUNOTOXICITY FINGERPRINT AT THE BIO–NANO INTERFACE—EMERGING IN SILICO TOOLS

Precise prediction of the interaction, distribution, and consequences of NPs in biological systems is essential for developing suitable NMs for medical applications and particularly for environmental and medical risk assessment of those. Conducting such a risk assessment based solely on experimental analyses is very time‐consuming and costly. Moreover, ethical considerations regarding animal testing must be taken into account. To circumvent these drawbacks, while complementing and accelerating experimental NM risk assessment, in silico prediction approaches to model NP–protein interactions and the resulting outcomes have been developed extensively in the last years (D. A. Winkler et al., 2014). To model the bio–nano interface with in silico tools, physics‐based methods like molecular dynamics, Monte Carlo, or coarse‐grained simulations are very promising (Dove, 2008; Latour, 2014). For example, the interaction of pristine NPs with biomembranes has been successfully predicted by the use of Monte Carlo and molecular dynamics simulations (Ding & Ma, 2015). Power et al. developed a multi‐scale modeling tool that directly relates physicochemical characteristics of NPs to protein binding affinities, to predict the composition of the protein corona, as molecular initiating event, by using physics‐based simulations (Power et al., 2019). At this stage, the most successful computational models, however, are data‐driven models like quantitative structure–activity relationship (QSAR), structure–activity relationship (SAR), or multi‐variate models. These methods use statistical and machine‐learning algorithms to predict correlations between the physicochemical characteristics of a NM, like size, shape, chemical composition, surface coating and charge, and their biological effects (Le et al., 2012; David A. Winkler, 2016). For example, QSAR approaches successfully predicted biological effects of NMs like the induction of protein adsorption, uptake, and cellular apoptosis by using datasets of biological assays investigating the distribution and NP‐induced toxicity together with nanodescriptors generated from extensive physicochemical characterization (David A. Winkler, 2016). Previous concerns regarding the applicability of QSAR models for complex materials like NMs, besides well‐established models for small organic molecules, have vanished to a great extent (Gajewicz et al., 2015; Le et al., 2012). For example, biological endpoints relevant for ecotoxicology (toxicity on various bio‐indicators like zebrafish, microalgae, or planktonic crustacean) of NMs have been modeled previously (Kleandrova et al., 2014). The cell association of NPs with their protein corona was predicted by using the Protein Corona Structure–Activity Relationships (PCSAR) model that relates cell association with the physicochemical descriptors developed for each protein in the protein corona (Kamath et al., 2015). For example, the immunotoxicity of NMs was investigated via computational studies applying SiteMap (Halgren, 2009) and docking calculations to predict binding sites suggesting that the internal hydrophobic pockets of some toll‐like receptors might bind carbon nanotubes and fullerene derivatives resulting in an experimentally confirmed pro‐inflammatory response in human lung carcinoma epithelial cells (Turabekova et al., 2014).

Nevertheless, the bottleneck of developing data‐driven in silico models with high predictive power, remains the dependency on freely available, high‐quality data‐ and metadata sets supplemented with a broad range of NP characteristics and description parameters. Generating these datasets still faces various challenges, like the high heterogeneity of NM characterization data found in the literature, impracticality of underlying datasets for modeling purposes, or inadequate completeness and quality levels (Tantra et al., 2015). To address these obstacles, initiatives for suitable NM data curation are increasingly established (Marchese Robinson et al., 2016; Powers et al., 2015). For example, the Nanomaterial Data Curation Initiative (NDCI) evaluated the current state of NM data curation and proposed opportunities to improve the progress for the NM data community (Hendren et al., 2015). Moreover, the MIRIBEL (Minimum Information Reporting in Bio–Nano Experimental Literature) standards were proposed to improve reproducibility, promote meta‐analyses and facilitate in silico modeling approaches (Faria et al., 2018). The existing NM libraries were analyzed for their capability to be integrated within novel nanoinformatics approaches and if they can be used for the development of NM‐specific Integrated Approaches to Testing and Assessment (IATA) for human and environmental risk assessment. Especially addressing the needs of industry and regulatory agencies to accelerate the evaluation of NM exposure, hazard, and risk assessment and promote NM commercialization by implementing computational safe‐by‐design approaches. Afantitis et al. recently summarized the latest advances in five main fields of the nanoinformatics sector (dataset curation, toxicogenomics modeling, multi‐scale modeling, predictive modeling, and human and environmental risk assessment) and provided recommendations on how to improve their further implementation in future computational modeling tools and NM infrastructure (Afantitis et al., 2020). They identified issues of data quality, reliability, and accessibility and highly recommended harmonization and connection of all NM data platforms to facilitate easy access to curated data under FAIR principles (Wilkinson et al., 2016). Additionally, the collection and interconnection of available scientific tools for nanoinformatics in state‐of‐the‐art platforms was identified as the most important next step to provide high interoperability and data re‐use. Despite all these challenges and barriers that still need to be overcome, in silico approaches (physics‐based and data‐driven methods) are now being widely adopted and are showing great success in improving and accelerating NM risk assessment and the development of NMs for medical applications, complementing the increasingly accepted tools for high‐throughput‐screening and safe‐by‐design development of new molecular entities and for preclinical purposes. For example, QSAR modeling by the commercial software ADMET Predictor® (Simulations Plus Inc., Lancaster, CA) allows to estimate 175 properties of existing and modified molecular entities, covering key domains such as solubility, tissue penetration, and toxicity classification, thus, laying the foundation for integrated absorption, distribution, metabolism, and excretion (ADME) and physiologically based pharmacokinetic (PBPK) modeling within preclinical assessments. However, in the field of NM, applied approaches are less integrated. For instance, Hassanian et al. used a combined approach of experimental (SEM, TEM, UV–Vis, and fluorescence spectroscopy) and in silico investigations (molecular dynamics simulation) to assess the safety of zinc oxide NPs in a structural study on human serum albumin (Hassanian et al., 2021). Recently, Maleki et al. provided a promising perspective on Alzheimer's disease research by showing results from multi‐scale (physics‐based and data‐driven) atomistic and microsecond coarse‐grained simulations suggesting that amine‐functionalized covalent organic frameworks can prevent ß‐amyloid aggregation, considered as the main culprit behind Alzheimer's disease development (Maleki et al., 2021). Wang et al. performed density functional theory (DFT) computations and QSAR modeling to investigate the adsorption of organic pollutants onto boron nitride nanosheets and demonstrated the applicability of in silico QSAR models for high‐throughput prediction of adsorption equilibrium constant values and, hence, for environmental nanosafety assessment (Y. Wang et al., 2021).

**FIGURE 3:**
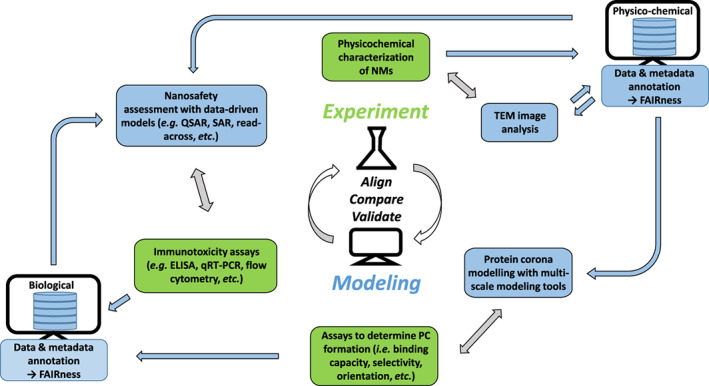
Schematic representation of an integrated experimental–computational workflow aligning, comparing, and validating in silico modeling by in vivo/in vitro assays (indicated by gray arrows). Data flows are depicted in blue arrows. Comprehensive metadata reporting and annotation results in data enrichment.

In order to further progress with these computational technologies, validation, and alignment of in silico and in vitro data investigating bio–nano interactions will be essential. This process could be facilitated by adopting an iterative exchange between experimentalists and modelers to identify the potentials and limitations of multi‐scale modeling tools and ultimately improve the predictability of computational methods as conceptually depicted in Figure 3 for which specific examples have become available recently.

To facilitate such interdisciplinary developments suitable interfaces are required. High‐quality data‐ and metadata (i.e., data about data; Papadiamantis et al., 2020) sets of the NPs' physicochemical characterization combined with results from in vitro investigations on protein binding and downstream biological effects may be uploaded to nano‐related data repositories, for instance, the NanoCommons Knowledge Base (https://ssl.biomax.de/nanocommons/), which provides access to a number of in silico tools and concomitantly facilitates data FAIRness (Findability, Accessibility, Interoperability, and Reusability; Wilkinson et al., 2016) by searching for the respective ontology terms (Hastings et al., 2015). For example, the NM image analysis tool NanoXtract (Varsou et al., 2020) calculates 18 image descriptors like circularity, convexity, diameter, and many more from an uploaded transmission electron microscopy (TEM) image, thus enabling fast generation of nanodescriptors and greatly enhancing the data extracted from one TEM image which can be used for further in silico modeling approaches. To enrich the data from in vitro experimentation, for example, protein binding experiments aiming to elucidate relevant parameters of protein corona formation, by in silico modeling a multi‐scale modeling tool called UnitedAtom has recently been developed (Power et al., 2019) to predict protein corona composition (adsorption energies and preferred orientations) by physics‐based atomistic simulations. For more advanced corona modeling, a hard‐sphere model called CoronaKMC has meanwhile become available as well, which considers the effects of protein concentration and size and accounts for competition between proteins (Rouse & Lobaskin, 2021). Moreover, improvement may be achieved by combining predictions from physics‐based models with predictions from data‐driven models like QSAR or multi‐variate models. These models have the ability to correlate NP characteristics and corona composition with cellular attachment and biological responses (Afantitis et al., 2018; Walkey et al., 2014). Altogether, integrated approaches of in vitro experimentation, physics‐based and data‐driven predictions, providing high‐quality data‐ and metadata sets for further investigations, will promote and accelerate nano‐related risk assessment and the safe and sustainable design (e.g., by digital twin‐based intelligent manufacturing [He & Bai, 2021]) of suitable NMs for medical applications and, in general, aid immunosafety of NMs.

## 5 PRE‐EXISTING CONDITIONS, IMMUNE EFFECTS, AND NANOMATERIALS

**FIGURE 4:**
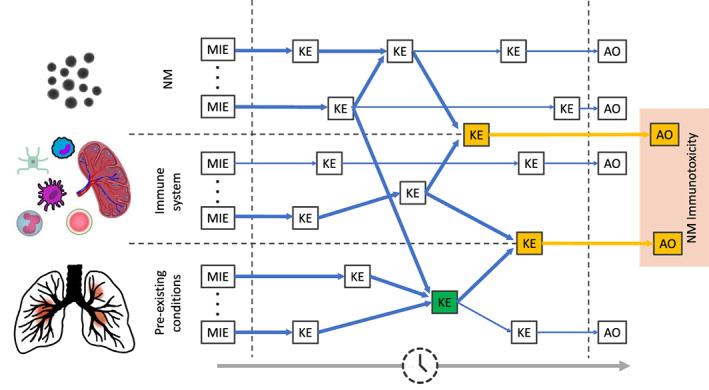
AOP immunotoxicity supra‐network, represented by new emerging and modified KEs, as a result of cross‐linking of NM, immune system, and pre‐existing condition AOP networks. MIE, molecular initiating event; KE, key event; AO, adverse outcome. Blue and orange arrows indicate key event relationships. KEs, AOs representing new emerging cross‐linking effects are marked in orange color, KEs depicting modifying cross‐linking effects in green color.

The term “immunotoxicology” was first coined in the 1970s and established a sub‐discipline of toxicology, focusing on the adverse effects on immune function that result from exposure to xenobiotics, notably chemical substances. This conception can be extended to particulate matter such as NMs, however, immune derangements are expected to be modified by adjuvant properties of NMs and new effects will occur (Chang, 2010) as a result of the NMs' physicochemical properties, noticeable their high surface‐to‐mass ratio linked with surface reactivity. The investigation of NM immunotoxicity may be a specific challenge (Marina A. Dobrovolskaia et al., 2016) if the effects of pre‐existing conditions are taken into consideration. A pre‐existing condition may be understood as any illness or the particular health condition of susceptible populations that exist at the time of NM exposure. At least one pre‐existing condition is prevalent in more than 50% of the adult population (Huguet et al., 2019) and each individual is affected more than once in a lifetime. However, while NM toxicology testing strategies are a matter of continuous advancements (Dusinska et al., 2017; Fernández‐Cruz et al., 2018), though also still full of pitfalls (Azhdarzadeh et al., 2015; Hofmann‐Amtenbrink et al., 2015; Warheit, 2018), the particularities of pre‐existing conditions in susceptible individuals are not thoroughly considered. As an example, a literature database search reveals that the term “immunotoxicity AND (pre‐existing conditions OR susceptible) AND (nanoparticle OR nanomaterial)” delivers a single‐digit number of specific research reports addressing this research area, exposing it as an orphan research topic. This fact may be due to the hyper‐complexity arising from cross‐linked AOP networks of NM, disease context, the role of the immune system and the spatio‐temporal dependency in vivo, which is conceptually outlined in Figure 4.

**FIGURE 5:**
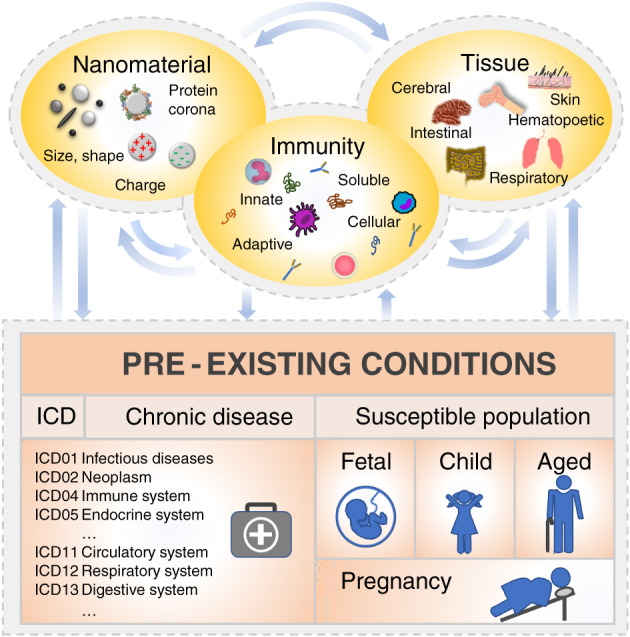
Immunotoxicity toward NM is influenced by a supra‐network of interacting AOPs, which is based on NM characteristics, the peculiarities of tissue environment, adverse effects on the immune system, and differences in susceptibility due to individual pre‐existing conditions. The latter may either result from the pathophysiological context of chronic disease or from the specific physiological context of a developing or aging individual and may give rise to modified immune system functioning when compared to a healthy adult state in the context of NM exposure.

Overall, on an individual's level, NM immunotoxicity can thus result from a supra‐network of interacting AOPs which depends on (1) the specificities of the NM's bioreactivity, (2) the peculiarities of the tissue microenvironment, (3) adverse effects on the entities of the immune system, and (4) pre‐existing conditions, as conceptually depicted in Figure 5. Pre‐existing conditions may either result from pathophysiological chronic disease or from the specific physiological context of a developing or aging individual. Pre‐existing disease conditions could be acute or chronic in nature; however, most acute pre‐existing conditions will resolve expeditiously and the chance of NM‐imposed additional adverse effects by environmental or occupational exposure is low. This could be quite different in chronic disease where persistent pathophysiological conditions provide a molecular, cellular, tissue, or organ context giving rise to newly emerging or modified AOPs, including aggravating effects (Figure 4, colored KEs), for example, by environmental soot on oxidative and inflammatory stress pathways in respiratory, cardiovascular disease and cancer (Niranjan & Thakur, 2017). Pre‐existing susceptibility may arise from physiological effects on maternal health during gestation, as well as particularities in an individual's development stage, such as fetal, neonate, childhood, or age. It may be obvious that such susceptible individuals are affected differently due to compromised immune functions. For example, in multiple sclerosis patients, gestation is a naturally occurring disease modifier with a significant reduction in relapse rate as a result of a reduced lymphocyte activation (Spadaro et al., 2019). Impaired capacity of self‐repair and tissue homeostasis due to stem cell aging (Schultz & Sinclair, 2016) by damage accumulation is reported for multiple tissues and organs, including, but not limited to the hematopoietic system, intestine, brain, and skin. Physiological alterations in ADME of NMs additional to unclear NM ADME behavior in vivo (A. Zhang et al., 2020) hinder the effective NM tissue dose prediction and dose bridging to in vitro test systems, and is a major obstacle to the clinical translation of ex vivo determined NM immunotoxicity effects.

### 5.1 Chronic diseases and immunotoxicity

The international classification of diseases (ICD; WHO, 2022) allows a systematic screening for diseases, thus supporting the selection of showcases where susceptibility to NM exposure in specific tissues could concur with a chronic disease state and immunotoxicity effects should be expected. To elucidate the challenges and pitfalls in the investigation of NM immunotoxicity in the light of pre‐existing conditions, we synthesized two such showcases considering perspectives of immunology, NM toxicity, and the specific disease properties. We highlight infectious diseases, which could be aggravated by NM and neoplasm, where NM could provide an enabler function for disease progression.

#### 5.1.1 Infectious diseases

The starting point of an infection in healthy individuals is making contact with outer and inner epithelia most prominently with bacteria or viruses. Most bacteria are not pathogenic but contribute to a persistent, phylogenetic complex (Grice et al., 2008) and species‐rich microbiome (D'Argenio & Salvatore, 2015). The microbiome is essential for human health and physiology and is, as such, vital for the induction of immune tolerance (Honda & Littman, 2016), which, when lost, may immediately have severe health consequences by aggravation of autoimmunity (De Luca & Shoenfeld, 2019) or inflammation and loss of epithelial functionality by tissue damage.

The epithelia of the lung extend to a surface area of 30–100 m^2^, depending on the phase of the respiratory cycle (Fröhlich et al., 2016), and those of the digestive tract 30 m^2^ (Helander & Fändriks, 2014) and are therefore perceived as the main hazardous routes for NM exposure. However, the skin, with just 1.7 m^2^ in surface area, may have the most intense exposure to NM per surface area unit (Larese Filon et al., 2015). NM exposure there occurs intentionally by use of life‐style products, such as sunscreen, or unintentionally by occupational or environmental exposure. Although the healthy skin provides a highly effective barrier, it may allow the penetration of NM to the dermal layer by diffusion, but selective in its physicochemical properties, demonstrated in animal studies (Ryman‐Rasmussen et al., 2006). The situation is different in the dermal papillae of hair follicles, where NM can accumulate permissively upon exposure (Lademann et al., 2009; Larese Filon et al., 2015; Sadrieh et al., 2010) and skin penetration was confirmed in human volunteers (Tan et al., 1996). As an example, superficial bacterial folliculitis, caused by gram‐positive or gram‐negative bacteria, a frequently chronic condition in susceptible individuals, may coincide with significant NM exposure and interference in innate immune activation by live bacteria (Swartzwelter, Fux, et al., 2020). The observed effect of NM on bacterial stimulus exerted by lipopolysaccharide (LPS) to the innate immune response is downregulation (Li et al., 2016; Swartzwelter, Barbero, et al., 2020) in vitro with human primary monocytes and Au NPs or upregulation (Inoue et al., 2006; Lebre et al., 2018) ex vivo in mouse peritoneal macrophages and bone marrow‐derived dendritic cells with pristine graphene and carbon black NPs. ROS‐mediated amplification of apoptosis in liver macrophages and hepatocytes was observed with 10 nm Au NPs in combination with bacterial LPS in a mouse model and (Yang et al., 2022). Changed activation of myeloid cells by collaboration of involved membrane‐associated triggering toll‐like and soluble NOD‐like receptors by PAMPs is also a proposed mechanism of action (Matos et al., 2021). The particularities of NM may determine, whether a beneficial effect of increased macrophage activation, monocyte recruitment, and bacterial clearance may help to better curtail an inflammatory process, or whether an adverse outcome with a non‐resolving or excessive inflammation and subsequent tissue damage may occur.

#### 5.1.2 Neoplasm

Development of cancer is a process in which cells, by a multistep course of events, undergo progressive genomic alterations. At the tissue scale, the starting point is the formation of an abnormal cellular mass by increased mitotic activity. Neoplasm may be benign or malignant, the latter with morphologic features such as significant cytologic atypia, tumor cell necrosis, high proliferation rate, invasive growth pattern, and metastasis (Patel, 2020; Sinha, 2018). Neoplasm can be intrinsically benign with low progression rate to malignancy, and malignant neoplasm may include benign precursor stages, but the progression patterns exhibit large differences in tumor types. Of important note, the fate of tumor development is decided in the microenvironment of small tissue‐specific tumor‐originating niches (Buder et al., 2019). This may give rise to the hypothesis that even small alterations in such specific niches by NM‐induced biological effects on immune effector functions may negatively affect host tumor defense mechanisms and induce immunotoxicity. The hallmark of cancer development is based on accumulation of sequential mutational events (hits) and DNA‐damage over a longer time (Ashley, 1969; Tomasetti et al., 2014; Zhang & Simon, 2005) allowing cells to acquire malignant properties while bypassing the natural anti‐tumor response of the immune system and exploiting intra‐tumoral cellular competition of the fittest mutational patterns (Nunney & Muir, 2015). Mandatory genetic pathways are sustained proliferative signaling, mechanisms to resist apoptosis, achieving replicative immortality, evading growth suppression, and the activation of invasion and metastasis pathways by epidermal–mesenchymal transition (Hanahan & Weinberg, 2011). Building on this, NM immunotoxicity effects may contribute to an enabler function for tumor progression by tumor‐promoting chronic inflammation involving KEs of NF‐κB signaling, inflammasome signaling, tumor‐infiltration, and immune checkpoints in the tumor‐microenvironment (D. Hanahan & Weinberg, 2000; Taniguchi & Karin, 2018). Moreover, immunosuppressive mechanisms may hinder a defense response against tumor cells. These may originate from cytotoxicity and genotoxicity on immune effector cells, for example, natural killer (NK), natural killer T (NKT) cells of the innate immunity, CD4^+^ helper T, and CD8^+^ cytotoxic T lymphocytes of anti‐tumor‐directed adaptive immunity, and on antigen‐presenting cells (APCs), DCs within the tumor tissue niche (Bahrambeigi et al., 2019). Mechanistic studies unveil that certain NMs, such as multi‐walled carbon nanotubes induce cytotoxicity only, while polystyrol NPs additionally induce DNA‐fragmentation, and Au NPs and ZnO NPs reveal genotoxicity in early time points in an in vitro T lymphocytes model with Jurkat E6‐I cells (May et al., 2022). TiO_2_ NPs induced genotoxicity in peripheral blood mononuclear cells (Ghosh et al., 2010). TiO_2_ as well as ZnO NPs were found to be genotoxic to human lymphocytes (Khan et al., 2015). However, adverse effects of NM on immune effector cells may be not confined to the tumor tissue niche, but extend to remote secondary lymphoid organs, such as lymph nodes. A successful anti‐tumor immune response relies on the selection and activation of tumor antigen‐directed lymphocytes by APCs there. While the tumor antigens originate from the tumor tissue niche (Leko & Rosenberg, 2020; Vigneron, 2015), NM immunotoxicity effects could also occur in the spatiotemporal context of APC activation, migration to the sentinel lymph nodes, antigen presentation, selection, and proliferation of T lymphocytes. This is of particular importance because there is mounting evidence for NP rapid translocation to lymph nodes (Choi et al., 2010). Immune modulation by exploiting NM's adjuvating properties and enriching with tumor antigens in tumor‐draining lymph nodes reshaped the anti‐tumor response in C57BL/6 mice (S. N. Thomas et al., 2014). Anecdotally, accumulation of environmentally originating heavy metal NP in lymphoid organs was linked to direct effects of immunotoxicity and malignancy (Iannitti et al., 2010).

### 5.2 Susceptible populations

The particular vulnerability of susceptible populations, including fetal development and pregnancy, infantility, and the elderly, to effects of NM exposure, can be largely attributed to divergences in ADME of NM in these groups (Li et al., 2014), or to differences of the immune state, being actively downregulated, immature (Ygberg & Nilsson, 2012), or in progressive immune senescence (DiCarlo et al., 2009; Dowling & Hodgkin, 2009; Panda et al., 2009). NM–immune system interactions, in this context, may confer an inherently increased risk of adverse immune effects. However, immunotoxicity in these conditions is probably multicausal, integrating altered inhibitory, and activation pathways and culminating in disturbed immune homeostasis and entailing modified or new emerging AOPs (outlined in Figure 4). Phenomenological experimental systems may not be able to reproduce this network complexity, hence, in silico models like nano‐QSAR or PBPK modeling and genome‐scale metabolic network (GSMN) could come to rescue (Singh et al., 2020). Interacting AOPs in the context of immunosenescence in the elderly comprise, for instance, reduced NM clearance capacity, impaired chemotactic responses and migratory function of neutrophils (Fulop et al., 2004; Wessels et al., 2010), macrophage phenotype shift toward M2 (Jackaman & Nelson, 2014; Mahbub et al., 2012), impaired dendritic cell activation due to dysfunctional toll‐like receptor‐signaling and reduced phagocytic and migratory capacity (Agrawal et al., 2007; Gomez et al., 2005), and loss of naive T cells (Goronzy et al., 2015; Vescovini et al., 2014).

Age group‐specific ADME is not only a matter of age‐related comorbidities (Mangoni & Jackson, 2004) in the elderly, but also a matter of age‐related organ morphometry or age‐related changes in physiological functions (Li et al., 2014). Excretion and distribution in elderly with impaired renal clearance may be modulated in any direction, because the kidney filtration threshold for particulate matter (5.5–8.0 nm; J. Liu et al., 2013) may change.

In the respiratory tract, adsorption of NP is expected to be age‐specific with alterations upon aging, e.g., by loss of physiologic reserve due to reduced pulmonary vital capacity, increased residual volume or airway‐obstruction, and impairment of NP clearance by disruption of the function of alveolar macrophages (Tran et al., 2018). This changed respiratory physiology gives rise to alterations in flow rates and retention time of inhaled particulate matter, thus in deposition on the respiratory epithelia by diffusion, impaction, and gravitational settling (Tsuda et al., 2013). Indeed, in the context of inhalable multiphase aerosols, significant differences in particle adsorption and localization of deposition hotspots in the respiratory tract, due to age‐specific lung morphometry and breathing patterns, were identified by in silico methods (Hofstätter et al., 2021).

Pregnant women and their fetuses are orphan populations, not only in respect to the safety and efficacy evaluation of drugs or vaccines (Kazma et al., 2022), but also to adverse effects upon NM exposure, although developmental toxicity is extensively investigated in animal models (Li et al., 2014), however, with limited predictive coherence for humans and without considerations of immunotoxicology effects, given the complex and dynamic nature of pregnancy, fetal development, and NM real‐world exposure. For a developing fetus, the threshold for resilience to adverse effects and toxic developmental insults elicited by NM may be extremely low. Decidua and placenta, the interface between mother and the developing fetus, not only provide an immunological barrier providing maternal tolerance while maintaining innate immune responses, but also a physical barrier function (Ander et al., 2019), thus, may abrogate certain mechanisms of ADME and prevent any meaningful dose prediction along with the NM exposure route in vivo. NM may also elicit perturbations at this barrier by immunotoxicity interactions giving rise to impaired fetal development, for example, placental toll‐like receptors activation, restriction of placental growth, and impairment of placental hormones secretion (Dugershaw et al., 2020); or NM may translocate further to the fetus (Mortensen et al., 2019). Adverse effects of NM to maternal health may precipitate by soluble mediators, such as cytokines, to the unborn child. The decidua, placenta, and umbilical cord impart innate immunity by tissue‐specific phenotypes of leukocytes which may help to protect the fetus from intrusion of pathogens, such as virus particles (Hurtado et al., 2010), but also NM (N. M. Liu et al., 2021), the latter with an opportunity to bring a maternally inherited protein corona along, a recognized MIE in AOPs. Immunotoxicity effects in the placenta require in situ characterization of NM, understanding the persistence, stability, and biotransformation giving the NM its particular immunological identity. All available methods fail to provide a comprehensive characterization of NM in situ (Mortensen et al., 2019). Another pitfall in immunotoxicity investigations is the lack of reliable dose information, bridging NM exposure to an observed in vivo tissue dose, because ADME prediction considering the maternal‐fetal interface goes along with a high degree of uncertainty (Hecht, 2020). In this regard, lack of dose justification disqualifies the majority of available literature. Animal studies have to be interpreted with caution, because rodents, the most frequently used animal models, present a higher, labyrinthine surface area for adsorption compared to human's villous placental structure (Benirschke & Kaufmann, 2000). Bypassing the problems of animal testing, ex vivo investigation of human placentas confirmed pulmonary to placental translocation of inhaled complex mixtures of carbonaceous and metal‐bearing air pollutants in nano‐scale, from combustion and friction‐sources. Translocated particles were found in macrophage‐enriched placental cells of the umbilical cord, hence, leaving behind the decidual and placental barriers (N. M. Liu et al., 2021). Advanced in vitro co‐culture models have been applied to investigate the potential for translocation across placental barriers and to elucidate adverse effects on placental stem cells. Using 50 nm and 0.5 μm‐sized polystyrene (PS) nano‐ und microparticles in a multi‐endpoint mechanistic study revealed no potential for translocation and weak embryotoxicity (Hesler et al., 2019) which was in opposition to earlier ex vivo placenta perfusion model studies of PS particles (Grafmueller et al., 2015; Wick et al., 2010). As of now, the actual body of evidence concerning adverse immunologic effects of NP on fetal health is not conclusive. NP translocation across the placental barrier has been evidenced in vivo for the human placenta as well as NP clearance by tissue‐resident macrophages. Depending on NM, these could provide MIEs for AOPs. However, adverse effects on placental or fetal development in humans are still elusive.

## 6 NANOMATERIAL DOSE JUSTIFICATION: A LENGTHY RUNWAY IN THE INVESTIGATION OF IMMUNOTOXICITY, EVEN WITHOUT PRE‐EXISTING CONDITIONS

The immune system is a complex network of surveillance and effector functions that secure the integrity of an organism, thus, providing protection against infectious microbes, non‐infectious foreign substances, but also aberrant self‐structures, such as tumor formation. These functions are established by physically separated generative (bone marrow, thymus) and peripheral organs (lymph nodes, spleen) responsible for immune cell growth, maturation, and activation. Immune cells cannot only be found in defined structures of lymphoid organs, but also as circulating cells in blood and lymph, and as a resident, scattered cells, abundantly found in nearly all tissues. Ancillary humoral immune responses are mediated by circulating proteins, found in the blood or other body fluids. These are either part of the innate immunity, the complement system, or part of the adaptive immunity when antibodies are involved. Secreted cytokines and chemokines, either from immune or tissue cells, orchestrate immune responses. These may be locally confined, such as recruitment from monocytes by chemokines to the afflicted tissue, but they may also emerge as a remote effect, for example, by activation of organ‐specific immune axis signaling (Jacobson et al., 2021), coordinating immune responses during infection or food allergy or maybe also exposure to NM. This complex network of the immune system, in particular its typically observed spatial separation between activation and effector functions, is of special importance for a thorough investigation of a possibly detrimental NM immune modulation. Pre‐existing conditions there could be decisive enablers or inhibitors of AOPs.

### 6.1 From NM exposure to a first tissue‐effective dose in vivo

**FIGURE 6:**
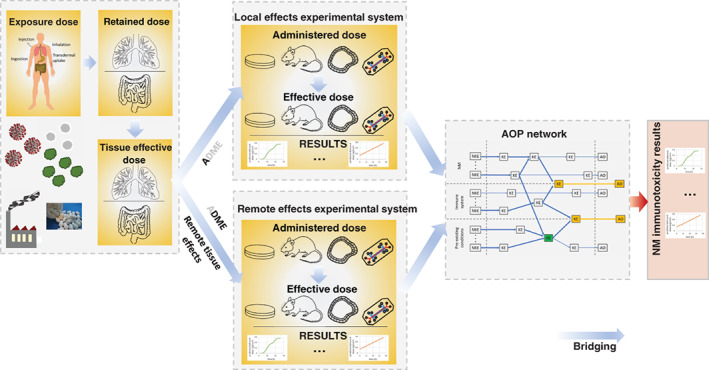
The multi‐step dose bridging process of NM exposure to NM immunotoxicity results. The design of a realistic, reliable experimental system on NM immunotoxicity requires a series of sequential dose bridging steps. NM immunotoxicity testing has to include experimental setups for local effect testing and additionally remote effect testing. The results can be finally linked to supplementary AOP networks in order to expand or assign relevance.

Irrespective of an immunotoxicity context, the evaluation of biological endpoints and the final translation to clinical relevance needs well‐reasoned bridging of meaningful real‐world human exposure scenarios into experimental models (Drasler et al., 2017; Gangwal et al., 2011; Schmid & Cassee, 2017) and supplementary AOPs (Figure 6).

The first physical contact of NM in a healthy individual or with pre‐existing conditions, starts virtually always at a protective barrier, either the skin, the respiratory, or the gastrointestinal tract. Non‐self‐recognition at and behind such barriers by professional immune cells is a common feature of an orchestrated immune response for the engagement of a vigorous defensive response or tolerance (Whibley et al., 2019) and is very conserved on all epithelia. In contrast, NM translocation to and adsorption by such epithelia, preceding an immune response, are highly variable, giving rise to pitfalls in the design of immuno‐ and genotoxicity assays and analysis and interpretation of readouts.

The dose, and more important the rate of adsorption determines the extent, but equally important the type of an immune response. Initial gene regulation upon NM recognition by professional immune cells occurs very fast, peaks in the first hours, and stipulates the final outcome (Kodali et al., 2013; Teeguarden et al., 2006). NM exposure via the skin (Larese Filon et al., 2015) or ingestion provides well‐accepted scenarios with approved applications such as lifestyle and healthcare products and as food additives, but both domains attain increased public awareness due to proposed new emerging safety issues around TiO_2_ in nanoform (Younes et al., 2021).

Experts link safety concerns of NP exposure predominantly to the inhalation route; ingestion and dermal uptake were in second and third place (Duschl & Windgasse, 2018). For the investigation of NM toxicology and respiratory health, dose justification is essential (Krug & Nau, 2017) and policy‐implemented environmental or occupational NM concentration limits could be set as a starting point, thus, providing some standardization and giving the experimental findings a real‐world significance. However, most submitted manuscripts are still deficient in this regard (Krug, 2014). Dose justification must not stop at the exposure level, it has to include a dose bridging from exposure to the effective dose at the tissue of investigational interest. Concerning the respiratory tract, most encouraging strategies could employ in silico methods to estimate a retained NP dose upon exposure. NP retention in the respiratory tract relies on the principles of the NPs' particokinetics, determined by the principles of diffusion, gravitational settling, impaction, flow rates, and airway geometry, to name the most important. Further, it depends on the morphometry of the lung as well as on the respiratory pattern (Hofmann, 2020). Employing in silico tools to determine a tissue‐effective dose is without alternative, but employing such tools requires comprehensive data on the physicochemical properties of the NM, which is frequently not available for the experimenter. Most in silico tools for NP deposition simulation in the respiratory tract are developed for the investigation of inhalation drug delivery, targeting the prediction of an overall retention fraction of the upper respiratory tract and neglecting the distal regions of the lung. However, regionalized NP deposition in the respiratory tract is a highly selective process, dominated by particle size (distribution) and density, local airway geometry, and flow rates. Hence, unequal regional NP deposition by a wide margin is to be expected and the identified NP deposition hotspots should pave the way for the identification of the appropriate dose for further in vitro investigation or in vivo with animal models. Several in silico tools are available and under development, the newer based on computational fluid dynamics (CFD), but CFD models are still regional only (Islam et al., 2020). Multiple Path Particle Dosimetry (MPPD), a more simplified model, allows to predict total, regional, lobar and local deposition in the whole lung, thus screening for deposition hotspots and estimating of the retained NP dose there (Anjilvel & Asgharian, 1995; Miller et al., 2016). All in silico models have their own limitations, but all models will fail in properly managing NM mixtures and the handling of other than log‐normal size‐distributions. In vivo, deposition hotspots may be linked to pathogenesis in the pulmonary tract (Hofer et al., 2021) and deposition preferences, for example, in the bronchial or bronchiolar region, may also establish or modulate pre‐existing conditions such as allergic reactions (Jarjour et al., 1997). Of additional note, among respiratory tract epithelia, there is a high variability concerning the NP elimination rate. Hence, a regionalized determination of the retained dose still does not properly reflect the potential for the induction of adverse effects. As an example, in the alveolar region of the lung a retained NP is adsorbed to a very thin fluid lining with a mean thickness of 200 nm (Knudsen & Ochs, 2018), and a translocation through this layer by stochastic diffusional mass transport is warranted within a very short time. The opposite is true when a NP is retained in the bronchial or bronchiolar region. Ciliated epithelia provide a highly effective outwards directed mucus transport with a velocity exceeding 1 mm/min in the bronchial region. The mucus layer provides a protein‐ and carbohydrate‐rich multi‐layer barrier of 10–20 μm (Atanasova & Reznikov, 2019), which can extend upon mucus hypersecretion in pre‐existing conditions, such as chronic obstructive pulmonary disease (COPD; Ramos et al., 2014). This reduces the probability of a retained NP being translocated to the epithelial barrier and to induce a response by immune cells or exerting adverse effects on epithelial cells.

Appropriate consideration of these differences in experimental settings, mimicking the air–liquid interface of respiratory epithelia, may be still more than a challenge and has to be addressed by advanced technologies better reflecting the (patho)physiology of such epithelia (Frijns et al., 2017). This is of particular interest, when inhalation of particulate matter may represent the KE of immunotoxicity by sensitization or reaction to bio–nano carriers representing, for instance, allergens (Himly et al., 2017).

### 6.2 Effective dose in vivo and its transition into the experimental system

The tissue‐effective dose determined in vivo is the starting point for dose consideration in any experimental system (Tsuda et al., 2013). A good coherence of biological endpoints, which are direct markers of an observed adverse effect, between both (tissue‐effective dose in vivo and tissue‐ or cell‐effective dose in the experimental system) should be expected, given that dose equivalence is established. Dose equivalence is achieved when the administered dose, that is the applied mass concentration in vitro or in an animal model, translates to an effective dose on a cellular level, matching the tissue‐effective dose determined in vivo. Closing this gap has been considered essential for years (J. M. Cohen et al., 2014; Paur et al., 2011). However, dose equivalence studies are still very rare, due to the high complexity and lack of easy to apply tools and methods.

In vivo as well as in vitro, particokinetics mainly determine the dose of NPs becoming effective on the targeted cells. As an example, the main driving mechanisms for a NP–cell interaction are diffusion and gravitational settling in a submerged cell culture system (J. M. Cohen et al., 2014) with adherent cells. At this point, comprehensive knowledge of particle characteristics in the presence of cell culture media, containing ions, proteins, and other molecular entities is inevitable (J. Cohen et al., 2013). The experimental determination of metadata, such as size distribution, density, charge, shape, agglomeration state, and stability over time is elemental input for dose considerations and the reproducibility of results (G. DeLoid et al., 2014; G. M. DeLoid et al., 2017; Krug, 2018).

A set of in silico tools is assisting in dose bridging for submerged cell culture experimental systems. These include In vitro Sedimentation Diffusion Dosimetry (ISDD; G. M. DeLoid et al., 2015; Hinderliter et al., 2010; D. G. Thomas et al., 2018) and its follow‐up version ISD3, Computational Fluid Dynamics (CFD) and Distorted Grid (DG). Polydispersity (Rodriguez‐Lorenzo et al., 2015) and agglomeration in physiological media, aggravates prediction of an effective dose, because it may lead to a broader size distribution by the build‐up of secondary particles with increased size, alterations of aspect‐ratio, and decreased density due to intra‐agglomerate accumulation of fluid content of the cell culture media. The importance of this agglomeration information was demonstrated by an in silico case study as part of a report on the significance of metadata to biological experiments (Papadiamantis et al., 2020). Of note, the effect of agglomeration may not be limited to alterations in the effective dose. It may additionally change the uptake behavior of phagocytic immune cells (Hirota & Terada, 2012; Leclerc et al., 2012), the gene expression, and secretion of cytokines, including chemokines, thus priming an immune response. Molecular entities of the cell culture media, in particular proteins and ions, are prone to affect particle stability and agglomeration behavior by corona formation. Hence, it is of importance for NP dose‐related effects as well as for modification in NP recognition due to its changed fingerprint, hampering predictive in vitro models (Jain et al., 2017).

Analogous to in vivo, decision making in vitro by professional immune cells about defensive or tolerance responses (Cao et al., 2017; Iwasaki et al., 2017) is performed in the very early stages of non‐self‐recognition and defines the subsequent biological endpoint. Hence, a high initial dose rate due to NP gravitational settling and subsequent dose rate variability is even more important to be considered than just the accumulated effective dose over time and bears the risk of cryptic overdosing (Himly et al., 2020).

Another challenge in dosing is the interpretation and consideration of the temporal behavior of the NP‐cell interaction. That is one reason why chronic exposure scenarios cannot be mimicked in vitro by simply administering a high initial NM bolus dose, merely creating overloading effects and non‐physiological responses, hence, missing the effects of long‐lasting inflammation, such as lung fibrosis (Keller et al., 2014; Shvedova et al., 2008; X. Wang et al., 2015). All attempts of acute/sub chronic to chronic exposure extrapolation are a source for major uncertainty (Dourson et al., 2021). This limitation of routine cell culture may be overcome by advancing to new techniques, such as 3D cultures, spheroids, organoids, and organ‐on‐a‐chip models, which better support the need for integration of long‐term incubation, human cell sourcing, and the organ‐specific microenvironment.

A frequent pitfall in NP toxicity evaluation, and particularly in immunotoxicity testing, is the missing decision on the most appropriate dose metric, rendering achieved results incomparable to the body of literature and incompatible for bridging existing AOPs. Most actual studies still report on mass concentration of NPs, which may be classified as the most inappropriate NP dose metric (Oberdörster et al., 2005, 2007). The effects that NPs exerted on immune cells, professional, and nonprofessional, are predominantly surface‐area associated: (i) induction of receptor‐mediated recognition and signaling, (ii) surface reactivity, (iii) release of toxic ions, and (iv) cellular compartment membrane damage due to particular physicochemical properties, such as, for instance, a crystalline structure (Nakayama, 2018). Under this premise, it is obvious that the applied NP surface area would contribute most to biological endpoints in immunotoxicity assays. This was confirmed by a meta‐analysis of particle‐induced pulmonary toxicity with different types and sizes of NM. The meta‐analysis concluded that 80% of the observed variability in toxicity findings could be explained by differences in the applied dose by surface area (Schmid & Stoeger, 2016).

The challenges and pitfalls discussed so far concern local immunotoxicology effects, where a NM exerts an adverse impact on immune cells, their embedding tissue, or the microenvironment. However, adverse effects may occur remote and distributed in vivo. Such effects may result from (i) the translocation of the NM to distal tissue and organs, (ii) distal immune modulation by soluble mediators as response to the local NM challenge, and (iii) adverse effects of locally primed and circulating immune cells on remote tissue. The investigation of translocation effects entails further considerations and methods, such as ADME. While in the local experimental context, adsorption largely defines the appropriate dosage regimen, in the remote context, distribution, metabolism, and excretion of NM have to be considered. As an example, NM adsorbed to epithelia of the lungs and penetrating to the interstitial tissue would be translocated to the sentinel mediastinal lymph nodes as demonstrated in rat models (Choi et al., 2010). Available in silico ADME tools may miss such a case, hence, for NM dose justification additional in vivo experiments may be inevitable.

Distal immune modulation effects via locally induced soluble mediators, such as pro‐inflammatory cytokines, may be of particular importance in the context of pre‐existing conditions, for example, as a disease modifier in the course of aggravation in autoimmunity or inflammation. For example, the IL‐1 signaling pathway is frequently found upregulated upon NLRP3 inflammasome activation and represents a KE of a NM‐induced inflammatory response. IL‐1 signaling, however, has also a pivotal role in the pathophysiology of autoinflammatory skin diseases, for example, psoriasis, and appears to contribute to disease progression by amplifying the inflammatory cascade (Cai et al., 2019). The thorough investigation of such remote effects requires the development of advanced in vitro or in vivo disease models. Alternatively, established AOP networks may help to identify such immunotoxicity effects.

Eliciting remote effects of locally primed and circulating immune cells in remote tissue is a basic principle of immunity. Thus, priming of immune cells, induced by or in the presence of NM, may include upregulation of anti‐inflammatory genes involved in tissue repair with a long‐lasting increased accessibility of chromatin by epigenetic features. Persistent chromatin modification in circulating monocytes has been observed 6 months after viral infection (Utrero‐Rico et al., 2021), but NM could also impart polarization, hence, affecting their physiological role in tissue homeostasis or disease. Several studies demonstrate that NPs can give direction to the polarization and the immunological function of macrophages (Miao et al., 2017).

### 6.3 Bridging to AOP networks, identifying new emerging/modified KEs, AOPs, and data gaps

AOPs organize biological and toxicological knowledge, for example, biological plausibility, quantitative understanding, time‐ and dose–response concordance, and documented evidence in a community‐curated manner. Effective exploitation and full realization of possible adverse implications of dose–response relationships in the experimental systems and associated biological endpoints, mechanistic, or phenomenological ones, can be achieved given that involved KEs can be successfully integrated into the growing body of established AOPs. Immunotoxic responses to stressors may escape identification by simply following a trajectory of an AOP downstream to its final outcome. Perturbations of a biological complex system are frequently the result of a synthesis of KEs of independent pathways, hence, the AOP single path approach needs an extension to a network concept (Knapen et al., 2018; Villeneuve et al., 2018). This is particularly relevant for the investigation of adverse outcomes in distributed systems, like the immune system, where interconnected AOPs may frequently share one or more KE(s).

The AOP framework made considerable advancements in the past years, representing a comprehensive network of AOPs relevant to toxicology and risk assessment. There is an increasing number of AOPs and MIEs where NMs act in the role of the inducing stressor. Moreover, the AOP framework provides a significant number of KEs which are concurrently associated with biological responses to NMs and with well‐defined associated adverse outcomes outside of the NM context. This dual attribution of KEs can be exploited to identify potential adverse outcomes which are out of reach of the experimental system for the investigation of NMs. For example, established AOPs list increase in reactive oxygen and nitrogen species, lysosome disruption, cytokine release, inflammatory cells infiltration, and secretion of pro‐inflammatory mediators as KEs in different AOPs with mutagenesis at the cellular level or neoplasm as final adverse outcome on the individual's level. These KEs are also observed effects in bio–nano interactions with the NM being in the role of a stressor. Thus, bridging these to existing AOPs may contribute to a better understanding of the broader context at cellular, tissue, organ, or individual's level, support the identification of data gaps, and promote the design of future experimental setups and research questions (Halappanavar et al., 2021).

The AOP concept is a relatively new tool and as such afflicted by shortcomings. Some of these may become apparent when the concept is applied to the investigation of immunotoxicity, where involved immune responses are an orchestrated move, being strictly in time, engage multiple players, endorse interaction from remote, and exert effector function to distal tissue. As such, the AOP concept struggles to cope with large groups of events, which are condensed into single KEs, for example, oxidative stress or inflammation. Mapping of multiple hit events, for example, in oncogenic pathways, may be another issue, because these cannot be attributed to one MIE. Compensatory or counter‐regulation mechanisms and effect duration complicate downstream KEs in AOPs and may frequently be omitted (Leist et al., 2017).

## 7 DEMAND FOR IN VITRO/EX VIVO MODELS TO INVESTIGATE NANOMATERIAL IMMUNOTOXICITY

With the rapidly growing number of NM applications in past decades many worldwide and national initiatives were established to advance the understanding of NM safety and to promote standardization of testing methodologies. In the field of cancer diagnosis and therapy applications of nanoparticles, the U.S. National Cancer Institute launched the Nanotechnology Characterization Laboratory (NCL), which in particular developed assay cascades for nano‐immunotoxicology testing, based on a tiered approach, spanning impurity and sterility testing, in vitro hematotoxicity and immunotoxicity testing and bridging to in vivo immunotoxicity testing by animal models (http://ncl.cancer.gov; Dobrovolskaia & McNeil, 2013). A similar initiative was launched by the European Commission, the European Nanomedicine Characterization Laboratory EU‐NCL (https://cordis.europa.eu/project/id/654190) with the intention to bridge research communities in the field of nanomedicine (Halamoda‐Kenzaoui et al., 2019). However, in the light of the outlined multidimensional complexities that the concerted action of the human immune system brings along, a strong demand for more complex models, such as co‐culture systems, arises. What simpler, monocellular in vitro systems lack is the intercellular context, the interaction of the cell with its microenvironment, and the organismic context. On the other end of the gap in experimental modeling, there are animal models, upfront murine models, which albeit their tremendous power for investigating mechanisms display shortcomings in their translatability to the “real” situation in humans. At this point, systems immunology comes into place (Davis et al., 2017), demanding to evolve from the study of its parts to a broader and more integrated view of how those parts work together and reveal the higher‐order cooperativity of cell types and pathways. Due to easier access, human studies are focused on blood cells offered as surrogate models for healthy or diseased states, however, the circulatory system is not an immunological organ itself rather the conduit through which, together with the lymph system, the immunological processes are transmitted between the relevant organs. Therefore, it emerges as the next logical step to develop experimental models further in the direction of advanced co‐culture models, 3D spheroids (excluding extracellular matrix, ECM) and organoids (including ECM), and organs‐on‐a‐chip, where indeed significant progress has been noted during recent years (Farhat et al., 2021), even for the intersection between an immune function with more immune‐distant organs (Cipriano et al., 2022). Of note, this development exerts a major impact on drug development (Khalid et al., 2017; Ma et al., 2021) and the overall biotechnological industry workflow (Franzen et al., 2019). For modeling immunity, the use of ex vivo cultures, including explants and slices, may pave the way to bridge between in vivo animal models and in vitro cell cultures, and here again the two scenarios, healthy versus diseased state are of interest. For each state ex vivo cultures, microfluidic devices, and engineered models of tissue are required, and these have to include models effectively mimicking primary immune tissues, like bone marrow, thymus, spleen, lymph nodes, and vessels, as well as peripheral immune tissues at entry or effector sites upon NM exposure such as lung, gut, or the central nervous system (Hammel et al., 2021). Still, shortcomings listed include that most models focus either on tumors or infection, so rather narrow in regard to different disease scenarios, or incorporate a rather limited number of cellular stakeholders such as macrophages or T cells. It will be essential, therefore, to step ahead with primary and pluripotent stem cell‐derived organ modeling approaches and promote patient‐specific model building by including several different cell types. Having accomplished that we still need to connect the thus established organ models into their systemic multi‐organ network, allowing for an improved consideration of NMs' ADME and distinction of local versus remote effects, which will, for the time being, require a deep involvement of computational workflows interoperably linked into the experimental ones. Here, machine and deep learning approaches based on FAIR big data from omics‐based systems immunology workflows (I. R. Cohen & Efroni, 2019; Culos et al., 2020; Pertseva et al., 2021) will one day hopefully prove useful.

## 8 CONCLUSION AND RECOMMENDATIONS FOR FUTURE RESEARCH AND DEVELOPMENT

So where do we stand now in light of the challenges that the highly complex immune system poses, and can we say something valid about specific (susceptible/vulnerable) groups within our cohort? We have opened the next chapters and need to actively and well‐focused pursue the above‐raised objectives, which were:

- We need to gain a deep understanding of the functionality of the human immune system in its well‐organized, spatially distributed, and time‐resolved highly complex nature. On this ground, NMs can be perceived as modifiers of pre‐existing conditions, which will one day allow to apply a more personalized approach to vulnerable groups.
- We have seen that the emerging concept of AOPs is well suited for the evaluation of immunotoxicity of NMs and the underlying mechanisms at a molecular level. With an increasing number of established AOPs that address NMs as stressors this concept will develop its full potential. Most NM‐relevant KEs in the context of immunotoxicity we highlighted complement activation, NLRP3 activation, and DC maturation.
- Protein corona formation represents the most genuine MIE that is relevant for NMs. We displayed (in Figure 1) that NM‐intrinsic properties, such as curvature, topography, and functionalization may cause binding of PAMPs, such as endotoxin, or result in epitope accumulation versus masking depending on random versus non‐random antigen orientation. In addition, fold disruption of bound homolog proteins may create DAMPs. These MIEs might lead to KEs such as protein aggregation/fibrillation, complement activation, DC maturation, T‐cell activation or polarization, pro‐inflammatory mediator release, granulocyte or macrophage infiltration, mast cell or basophil degranulation, and so forth resulting in different AOs.
- Experimental assays addressing the nano‐bio interface for a profound investigation of protein corona formation and the underlying molecular processes have their limits, giving room for developments in computational modeling. Integrating experimental and computational workflows will facilitate data enrichment. In Figure 3, we displayed a few recently emerging examples being specific for application with NMs. Sets of nanodescriptors being derived from in silico TEM image analysis can feed into protein corona or QSAR modeling procedures, and thus supplement risk assessment based on in vitro or in vivo experimentation. Integration of both worlds, the experimental and computational, will help to enhance the predictability of modeling procedures.
- Based on these conceptual considerations relevant for NMs, we dove into applying them on pre‐existing conditions, which indeed have a high prevalence in the general population. In the context of immunotoxicity of NMs and susceptible individuals, this research area clearly has an orphan status. Pre‐existing conditions can be ranging from chronic disease or particular development stages (fetal, pregnant, child, aged) alter immune functioning and ADME, thus interfere with NM's known biological effects in vivo. In combination, new AOPs may emerge, establishing an immunotoxicity AOP supra‐network.
- As noted in (i) the immune system is acting in a spatially distributed manner, orchestrated by mobile, scattered effector mechanisms provided by immune cells and soluble factors. Thus, a comprehensive understanding of NM‐related immunotoxicity needs to include the investigation of “remote” effects. This may have particular relevance in chronic disease states (e.g., infectious diseases, neoplasm) and susceptible populations.
- Remote effects testing strategies, however, need thorough elucidation of ADME and dose bridging in assay cascades in order to achieve meaningful clinical relevance. In silico methods, such as QSAR (nano‐QSAR) begin to unfold their potential in this field. However, scarcely available data still prevent meaningful dose prediction and dose bridging along the NM exposure route in vivo or fail at all to capture the specificities of pre‐existing conditions and susceptible populations.
- Finally, we touched upon the biological models and highlighted what their requirements would be to make them suited for resolving the bespoke well‐organized spatial distribution, which includes the microenvironment displaying the interaction and cross‐talk between cells. No matter what emerging cell model platform is applied (advanced 3D via spheroids to microfluidic‐based organ‐on‐a‐chip ones) limitations in disease scenarios exist. More can be expected from primary and pluripotent stem cell‐derived organ modeling and what remains it to establish the systemic multi‐organ networks enabling considerations, such as NMs' ADME and distinction of local versus remote effects.

Based on the laid out objectives we face thrilling times to come in NM‐related research, development, and innovation. By integrating experimental workflows with in silico modeling procedures we will be able to mutually enrich data, given we start sharing our datasets at measurable quality, supplemented with comprehensive metadata by FAIR means to effectively elucidate underlying mechanisms deploying the concept of AOPs. We furthermore have to acknowledge the diverse modifiers of immunotoxicity, presented by pre‐existing conditions, to be able to address the specific effects on susceptible individuals (humans are not a cohort of 100% similar lab animals or a homogeneous cell line). On the material side, a highly complex immunotoxicity modifier itself, we certainly need to start testing material mixtures (advanced materials are already around the corner). In order to realistically display in situ exposure conditions, and this holds true for in vitro models as well as for using lab animals, it represents a MUST to use realistic in situ‐delivered doses, for which computational modeling, such as PBPK, will be essential. Finally, regarding advanced cellular models, much still has to be developed in order to twin the spatial distribution of immune functioning with sophisticated 3D models.

## FUNDING INFORMATION

This work was supported by the international PhD program “Immunity in Cancer and Allergy ‐ ICA” of the Austrian Science Fund (FWF, grant W01213), the EU H2020 projects “NanoRigo” and “NanoCommons” (grants 814530 and 731032), the Production for the Future project “SmartCERIALS” of the Austrian Research Promotion Agency (FFG, grant 890610), and the Allergy‐Cancer‐BioNano (ACBN) Research Center of the PLUS.

## CONFLICT OF INTEREST

The authors declare no conflict of interest.

## AUTHOR CONTRIBUTIONS

**Sabine Hofer:** Conceptualization (equal); formal analysis (equal); investigation (equal); visualization (equal); writing – original draft (equal); writing – review and editing (equal). **Norbert Hofstätter:** Conceptualization (equal); formal analysis (equal); investigation (equal); visualization (equal); writing – original draft (equal); writing – review and editing (equal). **Benjamin Punz:** Investigation (equal); writing – original draft (equal). **Ingrid Hasenkopf:** Investigation (equal); visualization (supporting); writing – original draft (equal). **Litty Johnson:** Investigation (equal); visualization (equal); writing – original draft (equal). **Martin Himly:** Conceptualization (equal); formal analysis (equal); funding acquisition (equal); investigation (equal); supervision (equal); visualization (equal); writing – original draft (equal); writing – review and editing (equal).

## RELATED WIREs ARTICLES

Recent lab‐on‐chip developments for novel drug discovery

Nonclinical regulatory immunotoxicity testing of nanomedicinal products: Proposed strategy and possible pitfalls

## Data Availability

Data sharing is not applicable to this article as no new data were created or analyzed in this study.
